# The allocation of USdollar;105 billion in global funding from G20 countries for infectious disease research between 2000 and 2017: a content analysis of investments

**DOI:** 10.1016/S2214-109X(20)30357-0

**Published:** 2020-09-21

**Authors:** Michael G Head, Rebecca J Brown, Marie-Louise Newell, J Anthony G Scott, James Batchelor, Rifat Atun

**Affiliations:** a Clinical Informatics Research Unit, Faculty of Medicine, University of Southampton, Southampton, UK; b School of Human Development and Health, Faculty of Medicine, University of Southampton, Southampton, UK; c School of Public Health, Faculty of Health Sciences, University of the Witwatersrand, Johannesburg, South Africa; d Department of Infectious Disease Epidemiology, London School of Hygiene & Tropical Medicine, London, UK; e Department of Global Health and Population, Harvard TH Chan School of Public Health, Harvard University, Boston, MA, USA; f Department of Global Health and Social Medicine, Harvard Medical School, Harvard University, Boston, MA, USA

## Abstract

**Background:**

Each year, billions of US$ are spent globally on infectious disease research and development. However, there is little systematic tracking of global research and development. We present research on investments into infectious diseases research from funders in the G20 countries across an 18-year time period spanning 2000–17, comparing amounts invested for different conditions and considering the global burden of disease to identify potential areas of relative underfunding.

**Methods:**

The study examined research awards made between 2000 and 2017 for infectious disease research from G20-based public and philanthropic funders. We searched research databases using a range of keywords, and open access data were extracted from funder websites. Awards were categorised by type of science, specialty, and disease or pathogen. Data collected included study title, abstract, award amount, funder, and year. We used descriptive statistics and Spearman's correlation coefficient to investigate the association between research investment and disease burden, using Global Burden of Disease 2017 study data.

**Findings:**

The final 2000–17 dataset included 94 074 awards for infectious disease research, with a sum investment of $104·9 billion (annual range 4·1 billion to 8·4 billion) and a median award size of $257 176 (IQR 62 562–770 661). Pre-clinical research received $61·1 billion (58·2%) across 70 337 (74·8%) awards and public health research received $29·5 billion (28·1%) from 19 197 (20·4%) awards. HIV/AIDS received $42·1 billion (40·1%), tuberculosis received $7·0 billion (6·7%), malaria received $5·6 billion (5·3%), and pneumonia received $3·5 billion (3·3%). Funding for Ebola virus ($1·2 billion), Zika virus ($0·3 billion), influenza ($4·4 billion), and coronavirus ($0·5 billion) was typically highest soon after a high-profile outbreak. There was a general increase in year-on-year investment in infectious disease research between 2000 and 2006, with a decline between 2007 and 2017. Funders based in the USA provided $81·6 billion (77·8%). Based on funding per 2017 disability-adjusted life years (DALYs), HIV/AIDS received the greatest relative investment ($772 per DALY), compared with tuberculosis ($156 per DALY), malaria ($125 per DALY), and pneumonia ($33 per DALY). Syphilis and scabies received the least relative investment (both $9 per DALY). We observed weak positive correlation (r=0·30) between investment and 2017 disease burden.

**Interpretation:**

HIV research received the highest amount of investment relative to DALY burden. Scabies and syphilis received the lowest relative funding. Investments for high-threat pathogens (eg, Ebola virus and coronavirus) were often reactive and followed outbreaks. We found little evidence that funding is proactively guided by global burden or pandemic risk. Our findings show how research investments are allocated and how this relates to disease burden and diseases with pandemic potential.

**Funding:**

Bill & Melinda Gates Foundation.

## Introduction

Large amounts of funding are allocated to research in infectious diseases each year,^1^ spanning pre-clinical science, clinical trials, product development, and public health, including implementation research. These allocations involve numerous stakeholders across the global health community, including funders, researchers, policy makers, and clinicians.

However, there is little systematic tracking or detailed analysis of investments into research and development for infectious diseases to support how to make the best funding decisions.^2^ Nor is there systematic coordination between stakeholders involved in funding research and development, despite efforts such as the WHO Global Observatory on Health R&D to achieve better co-ordination.^1^

Funders differ in their approaches to commissioning research, from the curiosity-driven approaches of the Wellcome Trust,^3^ to the focused data-driven strategies of the Bill & Melinda Gates Foundation,^4^ which creates a heterogeneous landscape of research priorities. Thus, there is a need for an in-depth and comprehensive review of the global research and development landscape to identify what research has taken place, where the research was done, and which institutions were involved in the research. Such research on research is crucial for priority setting, informing funding decisions, and improving efficiency in allocating funds.^2^

**Research in context**

**Evidence before this study**

In November, 2019, we searched PubMed, internet search engines, and global health stakeholder sites, such as WHO, US Centers for Disease Control and Prevention, Wellcome Trust, Bill & Melinda Gates Foundation, and Policy Cures, using the search terms “research investments”, “research funding”, “infectious disease funding”, ”global health investments”, and “global health funding”, including only articles published in English. MGH searched a personal Mendeley literature database that includes published and grey literature concerning research funding. Previous investment analyses include Research Investments in Global Health study (ResIn) publications, the Policy Cures annual reports on product development research in infectious diseases, and numerous national reviews, for example the UK Clinical Research Collaboration annual Health Research Analysis of the UK research and development landscape.

**Added value of this study**

To our knowledge, this is the first study to describe in depth the global landscape for all infectious disease research from public and philanthropic funders. Our study covers 18 years of funding data, so captures long-term time trends and fluctuations. We combined and categorised awards using the classification system developed by the ResIn study. This strategy allowed us to provide a comprehensive overview of how infectious disease funding has been allocated, and compare findings with the global burden of disease, an important variable to consider when setting research priorities. This information can be used by global health research funders in decision-making processes.

**Implications of all the available evidence**

Our findings show that between 2000 and 2017, HIV received significantly more research funding than similar high-burden diseases such as tuberculosis, malaria, and pneumonia. The USA provides much of the global funding, particularly the US National Institute for Health. There are several infections that appear neglected compared with their burden of disease, such as syphilis and scabies. Thus, the global health community could use our findings to inform discussions, alongside other drivers for research prioritisation.

We present research done by the Research Investments in Global Health (RESIN) Study Group on research investments into infectious diseases from funders in the G20 countries across an 18-year time period spanning 2000–17, comparing amounts invested for different conditions and considering the global burden of disease to identify potential areas of relative underfunding.

## Methods

### Data collection

This study considered research awards related to infectious disease research from 987 public and philanthropic funders in the G20 countries (appendix p 2), made between Jan 1, 2000, and Dec 31, 2017. The methods used were similar to those described in detail elsewhere.^5^, ^6^, ^7^, ^8^ Data collected included study title, abstract, award amount, funder, and year.

Data were manually collated from multiple sources. Awards to institutions in the UK between Jan 1, 1997, and Dec 31, 2013, have been previously analysed.^7^, ^8^ Most data (>90%) from 2016 and 2017 were sourced from the UberResearch Dimensions database, which includes 4·9 million financial awards across health and non-health research and development sectors from 501 global funders. US National Institutes of Health (NIH) data from between Jan 1, 2000 and Dec 31, 2015, was sourced directly from the Project Reporter database. Other data were sourced from the websites of individual funders, funder databases such as the World Report, the UK National Research Register (a now-archived website owned by the UK Department of Health), or by contacting the funder directly and requesting data.

### Data analysis

We applied keyword searches and filters (appendix p 2) to identify studies of human-related infectious disease. Awards purely focused on plant pathology or veterinary science were excluded, unless there was a clear zoonotic component. Excluded studies were manually reviewed to identify any false negatives. The included financial awards were individually scrutinised to assess their relevance to infection.

MGH assessed all financial awards for inclusion and categorised infection-related awards, applying keyword labels as appropriate (appendix pp 2–3). Secondary checks on included and excluded awards were made (by RJB and other), as per the study protocol.^7^, ^8^ 15 000 (15·9%) of 94 074 awards were double checked, with a review of the award inclusion in the dataset and the applied labels indicating the disease areas of the research. Where there was disagreement, study information was provided to a third co-author for consensus.

Research award amounts were adjusted for inflation in original currency and converted to 2017 US$ using the mean exchange rate in the award year. Award amounts were missing for 6072 (5·7%) of 94 074 awards, from 13 funders (appendix p 2). In these cases, estimates were made using maximum award amounts for that funding stream as per the funder's website, by asking principal investigators for an approximate or exact award amount provided, or by asking in-country researchers who had knowledge of the research and development landscape for typical award amounts. Datasets and analyses were circulated to all authors for review and comment.

Included award types comprised project and programme grants, fellowships, and pump-priming or pilot projects. Excluded award types were conference and infrastructure grants and funding focused on operational activities rather than research.

Labels applied to each award included pathogen, disease areas and specialty (eg, antimicrobial resistance, respiratory, oncology, and paediatrics), and type of science along the research continuum (pre-clinical, phase 1–3 clinical trials, phase 4 and product development research, public health [focusing on populations], and cross-disciplinary studies across multiple stages of the research continuum). We defined cross-disciplinary as a study that covered more than one stage of the research continuum (for example pre-clinical research that progressed to a phase 1 study). Antimicrobial resistance included antibacterial, antiviral, antiparasitic, and antifungal resistance. The diagnostics category included research into screening. Sexually-transmitted infections excluded HIV, which had its own category. Neglected tropical diseases were based on the WHO definition (as of Oct 23, 2019).^9^

Burden of disease data were sourced from the Global Burden of Disease study online tool.^10^ Disease burden data are reported from 2017 for all infectious diseases, and additional examples are presented using HIV/AIDS, malaria, tuberculosis, and pneumonia from years 2005 and 2011 (six-year time intervals during the period covered by the investments dataset). Measures of disease burden analysed were mortality, disability-adjusted life years (DALYs), and years lived with disability (YLD). Comparison between awards and their associated observed disease burden were made by calculating investment per mortality, DALY, or YLD observed. We computed the investment relative to the burden of infection using the following equation: cumulative research investment up to the year of burden measure or number of deaths, DALYs, or YLD at that timepoint. For example, for assessment of HIV DALYs in 2017, the sum of HIV research investment from Jan 1, 2000, to Dec 31, 2017 ($42·1 billion), was divided by DALYs in 2017 (54 446 184), to get an investment per DALY metric of $772. Descriptive statistics and Spearman's correlation coefficient were used to investigate the relationship between research investment and disease burden, using Global Burden of Disease 2017 study data.

We used Microsoft Excel 2016 for data preparation Stata SE version 16 for data analysis.

### Role of the funding source

The funder of the study had no role in study design, data collection, data analysis, data interpretation, or writing of the report. The corresponding author had full access to all the data in the study and had final responsibility for the decision to submit for publication.

## Results

The final 2000 to 2017 dataset included 94 074 awards for infectious disease research, with a sum investment of $104·9 billion (annual range 4·1 billion to 8·4 billion) and a median award size of $257 176 (IQR 62 562–770 661; table 1).

**Table 1:** Global investments for infectious disease research 2000–17, by type of science, disease area or comorbidity, disease or pathogen, and year of award

		**Number of awards**	**Percentage of total**	**Funding (US$ billions)**	**Percentage of total**	**Median funding (IQR), US$**	**Mean funding (SD), US$**
Total		94 074	NA	104·9	NA	257 176 (62 562–770 661)	1 115 368 (5 282 231)
Type of science							
	Pre-clinical	70 337	74·8%	61·1	58·2%	238 124 (59 718–665 220)	868 782 (3 312 616)
	Clinical trials phase 1 to 3	2440	2·6%	9·2	8·8%	1 036 448 (312 765–2 961 159)	3 783 039 (14 700 000)
	Phase 4 and product development	1327	1·4%	1·7	1·6%	270 149 (100 000–989 758)	1 252 575 (3 699 940)
	Public health	19 197	20·4%	29·5	28·1%	270 444 (62 155–1 035 850)	1 537 692 (7 601 680)
	Cross-disciplinary	773	0·8%	3·4	3·2%	589 476 (91 221–2 990 422)	4 408 444 (14 736 094)
Disease area or comorbidity							
	Antimicrobial resistance	4845	5·2%	3·8	3·6%	191 710 (52 869–539 858)	781 036 (3 852 335)
	Behavioural or social science	5112	5·4%	5·3	5·1%	349 160 (82 940–1 181 447)	1 038 915 (2 183 452)
	Cardiovascular	969	1·0%	0·6	0·6%	155 638 (52 090–486 666)	612 814 (1 364 824)
	Chronic respiratory	625	0·7%	0·7	0·7%	385 128 (114 265–876 590)	1 073 074 (2 695 925)
	Skin and soft tissue infections	1036	1·1%	0·5	0·5%	155 422 (50 000–453 216)	528 429 (1 584 877)
	Drug use and addiction	1339	1·4%	2·0	1·9%	608 996 (171 625–2 143 382)	1 484 758 (2 159 933)
	Enteric	9268	9·9%	6·5	6·2%	167 454 (49 890–495 448)	701 225 (2 375 207)
	Gerontology	314	0·3%	0·3	0·3%	292 985 (84 534–906 417)	935 053 (2 220 080)
	Health-care-associated infections	1963	2·1%	1·4	1·3%	169 350 (47 423–548 100)	724 103 (3 257 520)
	Hepatology	5083	5·4%	3·4	3·2%	156 443 (50 703–498 827)	660 781 (1 957 538)
	Mental health	235	0·2%	0·3	0·3%	523 201 (143 684–1 667 876)	1 126 032 (1 565 351)
	Neglected tropical diseases	5221	5·5%	4·1	3·9%	133 123 (44 880–470 796)	744 922 (2 988 796)
	Neurology	3389	3·6%	3·3	3·1%	358 106 (76 321–1 034 196)	964 268 (2 319 882)
	Obstetrics	1199	1·3%	1·9	1·8%	326 562 (80 943–1 003 175)	1 604 117 (6 460 262)
	Oncology	4018	4·3%	3·5	3·3%	179 238 (50 936–571 189)	869 635 (5 450 843)
	Ophthalmology	376	0·4%	0·3	0·3%	245 986 (59 474–918 772)	859 637 (1 632 761)
	Oral	1037	1·1%	0·6	0·6%	111 136 (39 063–469 742)	557 466 (1 556 505)
	Paediatrics	3690	3·9%	5·6	5·3%	251 997 (58 192–987 621)	1 522 432 (7 485 250)
	Prison	157	0·2%	0·2	0·2%	701 645 (189 046–2 167 162)	1 520 540 (1 977 350)
	Respiratory	15 998	17·0%	18·5	17·6%	277 916 (66 041–833 542)	1 157 587 (4 478 451)
	Sepsis	1490	1·6%	1·1	1·0%	212 161 (56 349–740 000)	752 826 (1 961 561)
	Sexually-transmitted	4584	4·9%	3·7	3·5%	133 404 (33 184–470 197)	815 105 (4 991 830)
	Urinary tract infections	523	0·6%	0·4	0·4%	264 977 (83 762–675 493)	695 571 (1 286 641)
Pathogen or disease							
	African trypanosomiasis	650	0·7%	0·8	0·8%	315 433 (137 206–827 480)	1 183 651 (3 643 510)
	Anthrax	518	0·6%	1·0	1·0%	597 256 (181 356–1 979 251)	1 867 789 (4 157 831)
	*Aspergillus* spp	446	0·5%	0·3	0·3%	180 468 (44 346–482 741)	606 110 (1 666 160)
	Buruli ulcer	40	0·04%	0·02	0·02%	279 544 (101 659–464 926)	611 745 (1 148 503)
	*Campylobacter* spp	543	0·6%	0·3	0·3%	226 047 (54 100–465 336)	490 094 (983 705)
	*Candida* spp	1195	1·3%	0·6	0·6%	148 165 (45 671–463 142)	541 166 (1 092 445)
	Chagas	666	0·7%	0·4	0·4%	51 385 (37 103–370 772)	539 175 (1 471 485)
	*Chlamydia* spp	840	0·9%	0·7	0·7%	293 083 (75 000–680 691)	890 560 (3 814 782)
	Cholera	547	0·6%	0·4	0·4%	237 436 (59 775–661 232)	789 786 (1 597 530)
	*Clostridium* spp	825	0·9%	0·7	0·7%	268 621 (54 173–622 877)	828 251 (1 874 362)
	Coronavirus	396	0·4%	0·5	0·5%	266 922 (58 552–923 845)	1 241 720 (3 568 383)
	Crimean-Congo haemorrhagic fever	45	0·05%	0·05	0·05%	350 278 (78 943–823 281)	1 196 039 (2 840 648)
	*Cryptococcus* spp	340	0·4%	0·3	0·3%	305 917 (51 385–1 019 130)	861 530 (1 488 494)
	*Cryptosporidium* spp	239	0·3%	0·2	0·2%	167 415 (45 888–558 922)	648 857 (1 183 859)
	Cytomegalovirus	971	1·0%	0·8	0·8%	303 033 (89 039–751 988)	834 792 (1 730 354)
	Dengue	1064	1·1%	1·2	1·1%	188 297 (50 000–645 390)	1 087 023 (4 936 135)
	*E coli* (Enteric)	1261	1·3%	0·7	0·7%	152 385 (41 622–443 635)	571 835 (2 667 729)
	*E coli* (urinary tract infection)	214	0·2%	0·1	0·1%	314 020 (118 071–654 046)	633 605 (938 300)
	Ebola virus	506	0·5%	1·2	1·1%	475 138 (118 066–1 810 583)	2 436 055 (8 595 062)
	Epstein-Barr virus	887	0·9%	0·6	0·6%	167 755 (485 006–608 195)	698 088 (1 730 021)
	Gonorrhoea	314	0·3%	0·3	0·3%	363 831 (95 463–1 134 027)	995 488 (1 962 463)
	Hepatitis A	57	0·1%	0·04	0·04%	162 420 (48 726–719 823)	658 099 (1 144 165)
	Hepatitis B	1769	1·9%	0·9	0·9%	85 500 (42 253–359 695)	518 637 (1 655 673)
	Hepatitis C	3054	3·2%	2·4	2·3%	204 678 (54 336–619 686)	790 637 (2 231 365)
	Hepatitis E	122	0·1%	0·04	0·0%	79 699 (47 758–255 310)	293 887 (723 976)
	Herpes simplex virus	873	0·9%	0·8	0·8%	318 030 (88 100–791 155)	878 981 (1 639 525)
	HIV/AIDS	21 403	22·8%	42·1	40·1%	436 241 (110 709–1 497 634)	1 963 488 (9 117 206)
	Human papilloma virus	1848	2·0%	1·8	1·7%	198 616 (51 385–616 615)	6 912 483 (956 756)
	Influenza	3920	4·2%	4·4	4·2%	244 943 (54 501–850 560)	1 113 531 (3 360 834)
	Leishmaniasis	1328	1·4%	0·8	0·8%	64 264 (38 656–358 861)	567 952 (2 405 102)
	Leprosy	293	0·3%	0·07	0·1%	52 090 (35 984–148 619)	248 623 (712 026)
	Leptospirosis	188	0·2%	0·07	0·1%	51 415 (39 132–259 724)	372 127 (793 357)
	Listeria	466	0·5%	0·03	0·03%	176 172 (54 058–484 203)	622 783 (1 136 613)
	Lyme disease	586	0·6%	0·4	0·4%	315 166 (102 650–726 303)	729 722 (1 329 885)
	Lymphatic filariasis	221	0·2%	0·4	0·4%	141 525 (75 000–453 183)	1 822 870 (7 519 838)
	Malaria	4437	4·7%	5·6	5·3%	337 462 (103 167–840 520)	1 272 258 (5 495 134)
	Marburg virus	154	0·2%	0·3	0·3%	671 443 (376 340–2 269 867)	2 234 082 (4 777 032)
	Measles	208	0·2%	0·3	0·3%	223 931 (45 191–948 556)	1 480 828 (4 522 464)
	Meningitis	777	0·8%	0·6	0·6%	262 555 (74 645–580 847)	740 224 (1 751 265)
	Norovirus	324	0·3%	0·2	0·2%	158 784 (45 220–449 648)	698 908 (2 054 341)
	Onchocerciasis	78	0·1%	0·2	0·2%	242 016 (75 000–1 603 700)	2 201 094 (7 872 353)
	Pertussis	259	0·3%	0·2	0·2%	204 902 (53 615–595 021)	793 653 (2 781 053)
	Pneumonia	2748	2·9%	3·5	3·3%	227 570 (59 492–720 346)	1 295 821 (2 562 365)
	Polio	200	0·2%	0·4	0·4%	401 766 (107 770–1 338 367)	1 985 642 (4 738 529)
	Poxviruses	235	0·2%	0·5	0·5%	589 175 (205 723–1 629 883)	2 179 542 (8 424 070)
	*Pseudomonas* spp	1285	1·4%	1·0	1·0%	232 455 (72 782–600 924)	800 226 (3 023 734)
	Rabies	205	0·2%	0·1	0·1%	80 380 (37 719–386 201)	518 215 (1 557 300)
	Respiratory syncytial virus	556	0·6%	0·7	0·7%	389 191 (66 697–967 294)	1 338 414 (5 915 870)
	Rotavirus	384	0·4%	0·3	0·3%	134 496 (44 924–487 928)	1 039 924 (2 415 672)
	*Salmonella* spp	1526	1·6%	1·0	1·0%	191 669 (51 228–554 356)	634 310 (1 720 494)
	Scabies	38	0·04%	0·04	0·04%	426 115 (348 938–782 107)	1 112 631 (2 769 472)
	Schistosomiasis	618	0·7%	0·4	0·4%	131 941 (47 323–458 392)	636 016 (2 408 202)
	*Shigella* spp	246	0·3%	0·3	0·3%	184 518 (53 005–739 913)	1 193 408 (4 627 473)
	*Staphylococcus* spp	2357	2·5%	1·4	1·3%	171 442 (49 588–494 635)	592 705 (1 657 958)
	*Streptococcus* spp	1826	1·9%	1·3	1·2%	51 253 (205 875–590 096)	742 304 (2 316 068)
	Syphilis	111	0·1%	0·08	0·1%	193 501 (60 049–588 229)	765 455 (1 412 399)
	Tetanus	59	0·1%	0·02	<0·01%	127 306 (41 216–418 000)	408 647 (774 471)
	Toxoplasmosis	554	0·6%	0·4	0·4%	184 921 (53 378–488 582)	626 259 (1 250 402)
	Trachoma	28	0·03%	0·08	0·1%	505 246 (75 000–2 357 353)	2 085 780 (3 463 632)
	Trichomonas	60	0·1%	0·1	0·1%	296 798 (120 867–682 461)	2 327 692 (13 000 000)
	Tuberculosis	5246	5·6%	7·0	6·7%	298 502 (82 120–885 678)	1 339 356 (6 090 944)
	Varicella zoster	161	0·2%	0·2	0·2%	157 611 (48 506–687 311)	956 010 (2 988 433)
	Yellow fever	118	0·1%	0·09	0·1%	310 704 (52 090–721 749)	757 240 (1 215 103)
	Zika virus	491	0·5%	0·3	0·3%	201 447 (50 000–441 324)	622 804 (2 292 758)
Year of awards							
	2000	2493	2·7%	4·9	4·7%	541 368 (154 604–2 039 726)	1 975 701 (4 905 516)
	2001	2500	2·7%	4·1	3·9%	419 108 (136 670–1 640 451)	1 646 680 (5 570 833)
	2002	2647	2·8%	4·4	4·2%	534 528 (167 086–1 688 222)	1 668 438 (4 341 362)
	2003	2961	3·1%	5·7	5·4%	499 151 (158 408–1 655 190)	1 940 271 (7 066 292)
	2004	3333	3·5%	6·7	6·4%	517 408 (181 445–1 412 842)	2 022 233 (10 100 000)
	2005	3712	3·9%	5·6	5·3%	381 815 (102 307–1 223 215)	1 500 354 (8 736 730)
	2006	4575	4·9%	8·4	8·0%	256 724 (51 128–882 079)	1 844 582 (12 700 000)
	2007	5425	5·8%	6·5	6·2%	244 299 (47 387–700 500)	1 207 562 (5 107 707)
	2008	5693	6·1%	6·2	5·9%	244 030 (50 946–824 902)	1 090 595 (3 376 268)
	2009	6858	7·3%	7·3	7·0%	233 610 (61 690–729 664)	1 061 866 (3 927 947)
	2010	6861	7·3%	7·4	7·1%	217 586 (54 412–731 519)	1 084 433 (3 430 572)
	2011	6087	6·5%	5·4	5·1%	179 185 (57 092–603 637)	799 136 (2 291 067)
	2012	6383	6·8%	6·5	6·2%	254 484 (72 884–672 866)	1 019 646 (4 778 698)
	2013	6335	6·7%	5·8	5·5%	206 890 (52 090–625 480)	908 186 (4 011 321)
	2014	6070	6·5%	5·4	5·1%	216 119 (51 385–604 136)	897 432 (4 015 122)
	2015	5056	5·4%	4·5	4·3%	352 279 (102 650–730 956)	895 873 (3 951 852)
	2016	8135	8·6%	5·6	5·3%	158 778 (45 741–514 817)	683 117 (2 113 889)
	2017	7961	8·5%	4·2	4·0%	158 146 (50 000–460 625)	528 099 (1 772 225)

*E coli*=*Escherichia coli*. NA=not applicable.

By type of science, pre-clinical research received $61·1 billion (58·2%) across 70 337 (74·8%) awards. Public health research received $29·5 billion (28·1%) from 19 197 (20·4%) awards (table 1). Phase 1–3 trials received $9·2 billion (8·8%) across 2440 (2·6%) awards.

Phase 1–3 awards had the largest median award size ($1·0 million, IQR 1·3 million to 3·0 million), compared with a median award size of $0·2 million for each of pre-clinical (IQR 0·06 million to 0·7 million), product development (0·1 million to 1·0 million), and public health research (0·06 million to 1·0 million; table 1).

Funding for virology was $62·9 billion (60·0%), more than twice the amount for bacteriology ($27·3 billion [26·0%]), almost six times that for parasitology ($11·5 billion [11·0%]), and almost forty times that for mycology ($1·7 billion [1·6%]). By product type, therapeutics research ($18·3 billion [17·4%]) received more investment than did vaccines ($16·0 billion [15·3%]) or diagnostics ($3·6% [3·4%]; appendix p 2).

HIV/AIDS was the pathogen or disease with the greatest amount of funding ($42·1 billion [40·1%]) across 21 403 (22·8%) awards (tables 1, 2). Funding for tuberculosis totalled $7·0 billion (6·7%) from 5246 (5·6%) awards, funding for malaria was $5·6 billion (5·3%) from 4437 (4·3%) awards, and funding for pneumonia was $3·5 billion (3·3%) from 2748 (2·9%) awards. Funding for Ebola virus-related research was $1·2 billion (1·1%); $0·8 billion (68·0%) of all Ebola virus-related research investment was awarded between 2014 and 2017, following the high-profile outbreak of Ebola virus disease in West Africa in 2014.^11^ Similarly, $0·3 billion of funding was allocated to research on Zika virus, of which 96·0% was awarded in 2016 or 2017 after the Zika virus epidemic.^12^ Of the $4·4 billion (4·2%) of funding for influenza, $2·0 billion (45·0%) was awarded in 2006–10, with the highest annual funding amount awarded in 2009 ($0·6 billion [12·8%]). There were global outbreaks of H1N1 influenza in 2005 (so-called bird flu) and 2009 (so-called swine flu).^11^

Funding for coronavirus-related research was $0·5 billion (0·5%) from 396 (0·4%) grants, with a median award size of $2·0 million (IQR 0·6 million to 2·9 million; table 1). The US NIH provided $381·0 million (77·4% of all coronavirus-related funding) and $365·5 million (77·4%) was awarded to USA-based research institutions. Pre-clinical research accounted for $467·4 million (95·1%). The years with the greatest investment were 2004 ($149·5 million; 30·5% of coronavirus-related funding), the year after the international SARS outbreak,^13^ and 2015 ($87·7 million [17·8%]), the year after an outbreak of Middle East respiratory syndrome was reported in Saudi Arabia and a revised case definition was produced by Saudi Arabia and WHO.^14^ Further coronavirus-related results are presented in the appendix (p 4).

**Figure 1:**
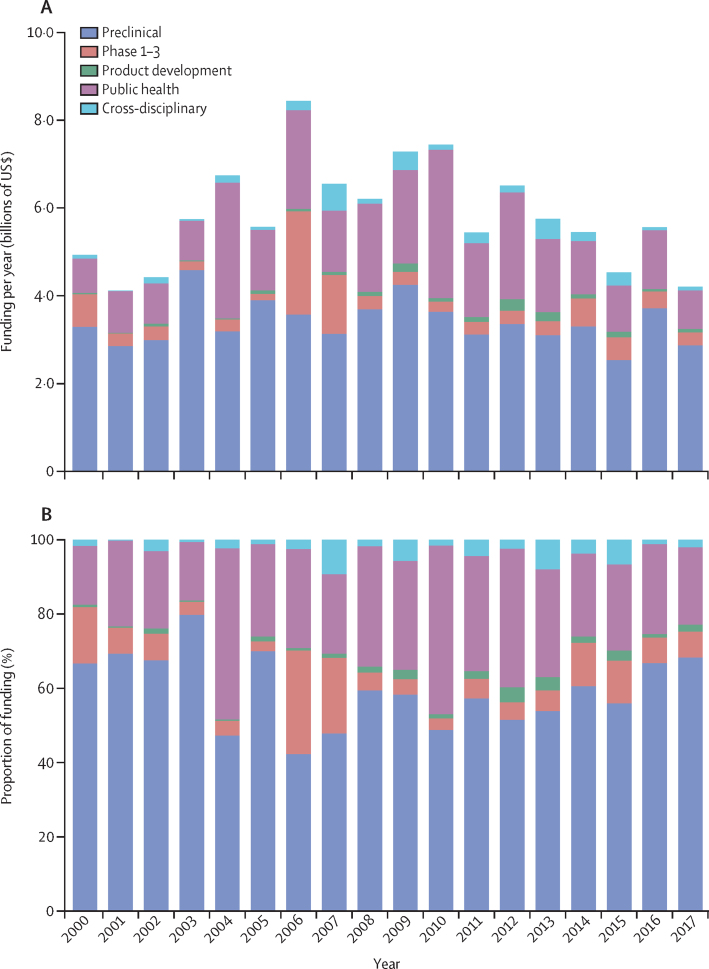
Funding per year by type of science (A) and proportion of funding per year by type of science (B)

By year of award, we found a general increase in year-on-year investment for infectious disease research between 2000 and 2006, but a general decline in the amount of annual investment between 2007 and 2017 (figure 1). Annual funding ranged from $4·1 billion (in 2001) to $8·4 billion (in 2006).

By disease area, $3·8 billion (3·6%) was awarded for antimicrobial resistance from 4845 (5·2%) awards, $4·1 billion (3·9%) was awarded for neglected tropical diseases, $1·1 billion (1·0%) for sepsis, and $1·4 billion (1·3%) for health-care-associated infections. In areas relating to hard-to-reach groups, $2·0 billion (1·9%) was awarded for infections related to drug use and addiction and $0·2 billion (0·2%) for infectious diseases in prison (table 1). Awards for comorbidities and non-communicable diseases included $0·3 billion (0·3%) for mental health and $0·6 billion (0·6%) for cardiovascular disease.

Funders from the USA provided $81·6 billion (77·8%) of the investment, which covered 42 926 (45·6%) of the awards. Within this, the US NIH was the largest funder, providing $59·4 billion (56·6% of total US funding) and the greatest number of individual awards (32 967 [35·0%]). The Bill & Melinda Gates Foundation provided $9·2 billion (8·8%) in 2317 (2·5%) awards. UK funders provided $8·3 billion (7·9%) in 8358 (8·9%) awards. When the awards had a clear geographical focus, $9·2 billion (8·8%) of the funding was focused on Africa and $2·4 billion (2·3%) on Asia (appendix p 2).

When ranking investment levels on the basis of burden of disease by DALYs across 34 infectious diseases (appendix p 2), African trypanosomiasis ($9740 per DALY) and genital herpes ($3101 per DALY) were ranked first and second, respectively (table 2). HIV/AIDS ($772 per DALY) was ranked eighth, tuberculosis ($156 per DALY) was ranked seventeenth, malaria ($125 per DALY) was ranked twenty first, enteric infections ($68 per DALY) were ranked twenty fourth, and pneumonia ($33 per DALY) was ranked twenty eighth. Scabies and syphilis were ranked joint last with $9 per DALY.

**Table 2:** Investment for selected infectious diseases compared with burden of disease, using 2017 disability-adjusted life-years

	**Funding, US$**	**DALYs in 2017**	**Funding per 2017 DALY**	**Ranking of funding per 2017 DALY**
African trypanosomiasis	$769 373 088	78 990	$9740	1
Genital herpes	$767 350 984	247 449	$3101	2
Leprosy	$72 846 615	31 513	$2312	3
Chlamydial infection	$748 071 028	355 096	$2107	4
Chagas disease	$359 090 978	232 143	$1547	5
Gonococcal infection	$312 583 361	303 103	$1031	6
Leishmaniasis	$754 241 255	774 211	$974	7
HIV/AIDS	$42 024 500 000	54 446 184	$772	8
Trichomoniasis	$139 661 501	242 814	$575	9
Dengue	$1 156 591 953	2 922 630	$396	10
Sexually-transmitted infections excluding HIV	$3 736 444 164	11 473 757	$326	11
Lymphatic filariasis	$402 854 250	1 363 953	$295	12
Yellow fever	$89 354 426	314 002	$285	13
Schistosomiasis	$393 057 872	1 431 447	$275	14
Trachoma	$79 259 634	302 919	$262	15
Rabies	$106 234 137	633 806	$168	16
Tuberculosis	$7 026 261 356	44 997 359	$156	17
Hepatitis C	$2 414 606 412	15 598 250	$155	18
Varicella and herpes zoster	$153 917 628	1 144 435	$134	19
Onchocerciasis	$171 685 350	1 342 937	$128	20
Malaria	$5 645 007 933	45 014 578	$125	21
Typhoid and paratyphoid	$967 958 038	9 800 988	$99	22
Urinary tract infections	$363 784 033	4 695 291	$77	23
Enteric infections	$6 498 954 989	95 209 183	$68	24
Hepatitis E	$35 854 299	738 508	$49	25
Measles	$308 012 254	8 156 526	$38	26
Hepatitis B	$917 469 643	25 282 942	$36	27
Pneumonia	$3 560 915 407	106 483 431	$33	28
Meningitis	$575 154 455	20 370 870	$28	29
Pertussis	$205 556 273	7 977 284	$26	30
Hepatitis A	$37 511 691	1 497 892	$25	31
Tetanus	$24 110 176	2 449 433	$10	32
Scabies	$42 279 961	4 528 672	$9	33
Syphilis	$84 965 600	9 909 025	$9	34

DALY=disability-adjusted life-year.

**Figure 2:**
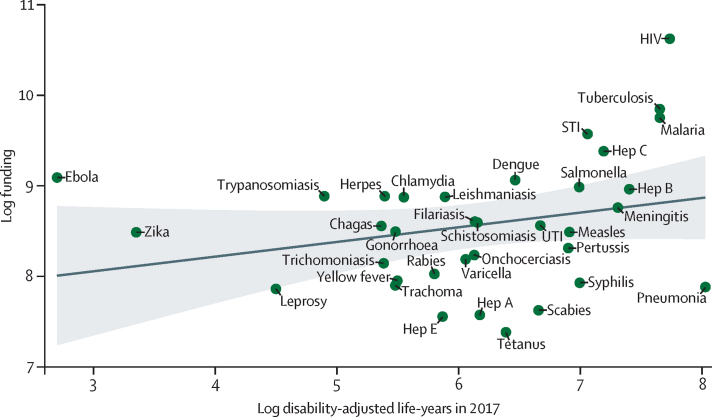
Association between investment and DALYs Filariasis refers to lymphatic filariasis. Trypanosomiasis refers to African trypanosomiasis. The line indicates fitted values and the shaded area indicates the 95% CI. STI=sexually transmitted infection. DALYs=disability-adjusted life-years. Hep=hepatitis. UTI=urinary tract infection.

When comparing investment for individual infections alongside 2017 DALYs, Spearman's correlation coefficient was 0·30 (p=0·048), suggesting weak positive correlation between research investment and global burden of disease (figure 2). Infections within the shaded area of the graph showed a stronger correlation between investment and burden of disease. Infections below the shaded area were relatively underinvested, and infections above the shaded area were relatively well-invested, compared with their 2017 DALYs burden.

When comparing investment by mortality, syphilis ($632 per death) and tetanus ($749 per death) were ranked the lowest of the 27 infections for which mortality data were available (appendix p 2). The highest-ranked infections by investment per death were those for which associated mortality is typically low, specifically chlamydia ($712 076 per death) and African trypanosomiasis ($563 094 per death). HIV was ranked seventh ($44 481 per death), malaria was ranked thirteenth ($9107 per death), tuberculosis was ranked fifteenth ($5936 per death), and pneumonia was ranked twenty fourth ($1392 per death).

**Figure 3:**
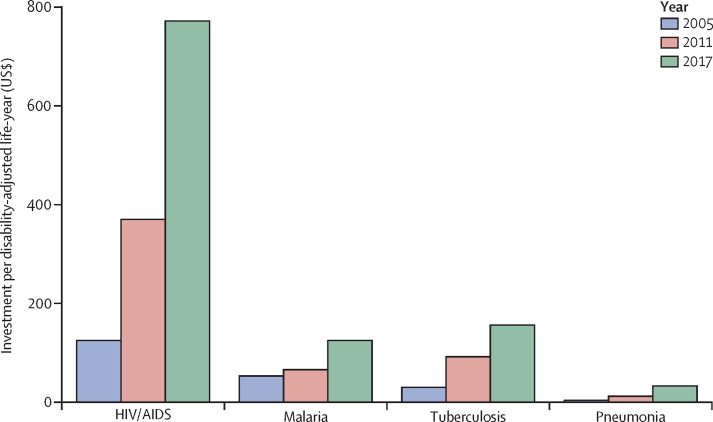
Research investment over time for HIV/AIDS, malaria, tuberculosis, and pneumonia, relative to investment per disability-adjusted life-years

Across different timepoints of the study, HIV-related research consistently received greater investment than did malaria, tuberculosis, or pneumonia (figure 3). Pneumonia-related research consistently received far less funding during the study period compared with HIV, tuberculosis, or malaria.

By type of science, 35·5% of research funding for HIV was for pre-clinical research, 15·1% for phase 1–3 trials, and 45·9% for public health research (appendix p 9). Pneumonia had the greatest proportion of funding allocated to pre-clinical science (55·7%) and the lowest amount for public health research (23·5%) compared with HIV, tuberculosis, and malaria (appendix p 2).

## Discussion

In this study, we provide an analysis of $105 billion of research investment as 94 074 public and philanthropic awards for infectious disease research covering the years 2000–17. Over half of this investment was for pre-clinical science and over a quarter for public health research. By type of infection, HIV-related research received more than double the investment for tuberculosis, malaria, and pneumonia combined. Infections that are relatively less well-funded include some sexually-transmitted infections (syphilis and gonorrhoea) and neglected skin infections, such as scabies.

Funding for coronavirus-related research was $0·5 billion from 396 grants, of which 95·1% was for pre-clinical research. However, in 2020 there has been a huge reactive effort to support the response to the COVID-19 pandemic, which includes substantial financing for research.^15^ As of Aug 4, 2020, the RESIN study has tracked $1·6 billion of global public and philanthropic research funding, which already exceeds the 2017 total investment in HIV research ($1·1 billion; appendix page 2). Viral respiratory infections are known to be one of the most likely causes of a pandemic, but despite this and the existing high levels of mortality in young children and older people due to such infections, systems for pneumonia research and advocacy are not well established.^16^ Confusion over the definition of pneumonia,^17^ few experienced researchers to make a strong case to funders, and few high-profile public figures championing the cause have led to pitifully low levels of funding compared with the disease burden. The Bill & Melinda Gates Foundation, which is guided by childhood deaths, is the main funder of pneumonia-related research.^5^

The metrics used to compare investment by burden of disease are misleading for pathogens such as Ebola virus and Zika virus, which at first appear to be relatively well-funded compared with their burden of disease (appendix p 2). However, for these conditions, which are public health emergencies, DALYs are not a fair metric to use. Outbreaks of this nature are not necessarily high-burden in terms of numbers of cases but are high-risk given the potential for rapid spread to cause widespread outbreaks, an important factor that influences research investment decisions. As illustrated by the evolving COVID-19 pandemic, there is a public health need to support outbreak responses and research should very much be part of such a response. Such outbreaks create uncertainty and fear, with media promoting a need to do something and urging political circles to respond rapidly.^18^, ^19^ Historical funding for coronavirus research was very low, even after the high-profile outbreaks of severe acute respiratory syndrome (SARS), due to SARS coronavirus, and Middle East respiratory syndrome and the potential for the rapid spread of such infections.^20^ Other analyses highlight how funding for neglected infectious disease research (distinct from neglected tropical diseases) is increasing.^21^ Our analysis supports this conclusion, for example, showing that research on neglected tropical diseases or with a focus on Africa is increasing (appendix p 2). There have been substantial declines in HIV funding, primarily in higher-income settings (and thus not captured under neglected disease definitions).

The Coalition for Epidemic Preparedness Innovations, founded in 2016, has received substantial research investment from multiple funders to research selected high-threat pathogens.^22^ For example, there are several ongoing studies to develop a universal influenza vaccine to reduce pandemic risk, as well as vaccine in development against coronaviruses.^23^ Antimicrobial resistance, which continues to be a serious worldwide threat,^24^ has led to the introduction of the Global AMR R&D Hub with a remit to address challenges and improve coordination and collaboration in global antimicrobial resistance research and development using a One Health approach.^25^ Antimicrobial resistance is also an important contributor to sepsis mortality, which is responsible for 11 million deaths annually with most of the burden in sub-Saharan Africa,^26^ but receives just 1·0% of the funding.

Research investment analyses can be a valuable audit of a system that has perhaps maximised scientific efficiency through peer review of curiosity driven research and provide a direction for revision of research on under-investigated diseases and subject-based opportunities. The COVID-19 pandemic has shown the fragility of national and global infrastructures, and pandemic preparedness will surely be a focus for high-profile global health research stakeholders in years to come. Sustainable tracking of how research funding is spent is vital to ensure that all priority areas and knowledge gaps are addressed,^15^ and there must be adequate translation of that new knowledge into policy and practice, with findings that can be feasibly adopted in resource-poor settings. Multiple factors other than the current and projected burden of disease influence research funding decisions, such as political drivers of decision making (notable in our study given the major funder was the US Government), advocacy and lobbying, emergency preparedness for emerging infectious diseases with pandemic potential, and public health research for conflicts and other humanitarian responses.

Applying a globally recognised label to a disease can be important. WHO oversees the designated list of neglected tropical diseases,^9^ which helps to raise the profile of these conditions and support arguments for research funding.^27^ As an example, African trypanosomiasis, which has been at the forefront of efforts to tackle neglected tropical diseases, has been described as an extraordinary success story, with a decline in the DALY burden by 93% between 2000 and 2017.^28^ African trypanosomiasis has received twice the amount of research funding compared with lymphatic filariasis or schistosomiasis, and more than non-neglected tropical diseases, such as meningitis or the respiratory syncytial virus. Researchers who study African trypanosomiasis have elimination and eradication in sight, although this will probably require further substantial investments.^28^ Investment in other neglected areas might help produce similar effective responses, although the type of research investment must be appropriate. For example, our analysis highlighted scabies and syphilis as particularly underfunded. Effective treatments are available for scabies, so the most useful research might be around an effective drug supply chain or addressing stigma.

Other factors beyond the burden of disease also influence the direction and amount of investment. The geographic focus of research investments affects the likelihood of knowledge being translated into policy and practice, particularly in the country or sector where the research was undertaken.^29^ It is important to consider where research capacity should be created or enhanced, rather than simply which research areas to prioritise and fund. The UK invests greater resources in former colonies, influenced by historical ties and a shared language.^30^ Investments in different sectors will also be affected by diplomatic considerations, for example, funding countries seeking cooperation from recipient countries or regions in response to security threats and terrorism.

This study has several limitations. There will be missing data, particularly when data could not be accessed from public and philanthropic funders. We propose that the effect of this should not be substantial and should not greatly influence our findings, as the included data relate to 18 of the top 20 leading investors in research.^31^, ^32^ The focus on G20 countries means that funders from countries who are not in this group but are proactive in global health research, such as Switzerland and Norway, were not included. A key challenge was integrating data that were presented in numerous different formats. Future analyses would be simplified considerably if funders could adopt a minimum dataset of required information, perhaps recommended by the WHO Global Observatory on Health R&D, which would require that applicants add standard labels (eg, the type of science along the research pipeline) to their project at time of submission. Applying categories to an award retrospectively is time-consuming and subjective, although errors were reduced by observations from a second author and consensus. Automated categorisation based on keyword searches is problematic, since the title and abstract of many awards contain references to diseases that are not the study areas of focus. Furthermore, separating out awards for operational or implementation research and activities that are non-research based implementation (ie, not generating new knowledge) is a subjective process. Our study lacks data from the private sector, particularly concerning tools and products such as vaccines, diagnostics, and therapeutics. The analyses use Global Burden of Disease study data, which are themselves modelled estimates and will on occasions be based on imputations due to missing data, and have been subject to criticism.^33^ Selection of timepoints for research investments and disease burden will affect our findings as both of these change over time.

More than $100 billion has been spent globally on infectious disease research between 2000 and 2017, but this funding does not correlate with current levels of burden or the level of risk posed by infections with pandemic potential. Since priority setting for research must consider many different factors, our analysis should be used to support decision making rather than providing clear-cut answers. It is worrying that the funding allocated to infectious disease research is declining during a period in which there are concerns surrounding antimicrobial resistance and the pandemic potential of many pathogens.

In conclusion, our findings show where research funding resources are allocated and how this relates to disease burden and diseases with pandemic potential. We anticipate that our results could be an invaluable resource to global health stakeholders (eg, WHO, research funders, or national governments) who define research strategy and make decisions about the allocation of restricted research and development resources.
