# Supplementary material for: The allocation of USdollar;105 billion in global funding from G20 countries for infectious disease research between 2000 and 2017: a content analysis of investments
Source: Lancet Glob Health. 2020 Sep 21;8(10):e1295–304. doi: 10.1016/S2214-109X(20)30357-0 (PMC7505652; doi:10.1016/S2214-109X(20)30357-0)
Supplement: Supplementary appendix [file mmc1.pdf]

THE LANCET
Global Health

Supplementary appendix

This appendix formed part of the original submission and has been peer reviewed. We post it as supplied by the authors.

Supplement to: Head MG, Brown RJ, Newell M-L, Scott JAG, Batchelor J, Atun R. The allocation of US$105 billion in global funding from G20 countries for infectious disease research between 2000 and 2017: a content analysis of investments. *Lancet Glob Health* 2020; **8:** e1295–304.

Appendix

Appendix number	Item	Page number
I	Webpage of extra data	2
II	RESIN classification system and definitions	3
III	Research investments related to Coronavirus 2000-2017, by year and type of science	4
IV	Research investments related to Ebola virus 2000-2017, by year and type of science	5
V	Research investments related to Zika virus 2000-2017, by year and type of science	6
VI	Donor investments by year	7
VII	Donor investments, proportion of funding by year	8
VIII	Research pipeline for HIV/AIDS, malaria, tuberculosis and pneumonia 2000-2017	9
IX	Investment for selected infectious diseases compared with burden of disease	10

Appendix I - Webpage of extra data

Please see www.researchinvestments.org/data for the following -

Further data on sum investment by i) microbiology (bacteriology, virology, parasitology, mycology, prion); ii) product (vaccine, diagnostic, therapeutic); iii) selected global regions (Africa, Asia, Oceania, South America, Central America and the Caribbean); iv) Funder country; v) Selected funders

Detailed breakdown of all disease areas

Breakdown of Coronavirus-related data

Further data on investment and burden of disease (mortality, and years lived with disability, YLD)

Keywords used for searches

List of funders

List of funders where estimates were made on the funding amount

Example studies

Flow chart of methodology

Appendix II - Definitions

Research

Our analysis considered only research awards related to infectious diseases, and excluded (for example) purely implementation projects.

In order to keep our analysis as straightforward as possible, our definition of research was an award that involved 'the creation of new knowledge and/or the use of existing knowledge in a new and creative way so as to generate new concepts, methodologies and understandings.'

This is a definition used by universities, for example https://www.westernsydney.edu.au/research/researchers/preparing_a_grant_application/dest_definition_of_research

Thus, to further clarify around the boundaries between research and implementation, any award that implements the findings of research was excluded. Any award that considered how best to assess and incorporate new approaches into a health system was classified as research.

We are of course happy to respond to individual requests for clarity about our inclusion and exclusion criteria.

Type of science definitions

- Pre-clinical – molecular, *in vitro, in vivo*, immunology, drug discovery
- Phase 1-3 clinical trials – includes RCTs, 'first-in-man' studies etc
- Phase IV, product roll out, pharmacovigilance,
- Public health – epidemiology, statistics, economics, social science, behavioural studies, population health, implementation research
- Cross-disciplinary – any project with significant components that encompass two of the above types of science e.g. pre-clinical research leading into a phase I trial

More on the RESIN study

The RESIN study has a new set of webpages, see the Clinical Informatics Research Unit website at https://www.the-ciru.com/resin

Also see the RESIN analysis around COVID-19 - https://www.the-ciru.com/resin-covid19

G20 countries

We selected the G20 countries as the scope for this study. This was in part due to the key funders main global economies being within the G20 and thus we can have a comprehensive analysis in place, and also a pragmatic decision around person-time available and what is realistically achievable.

Appendix III – Coronavirus R&D funding 2000-2017

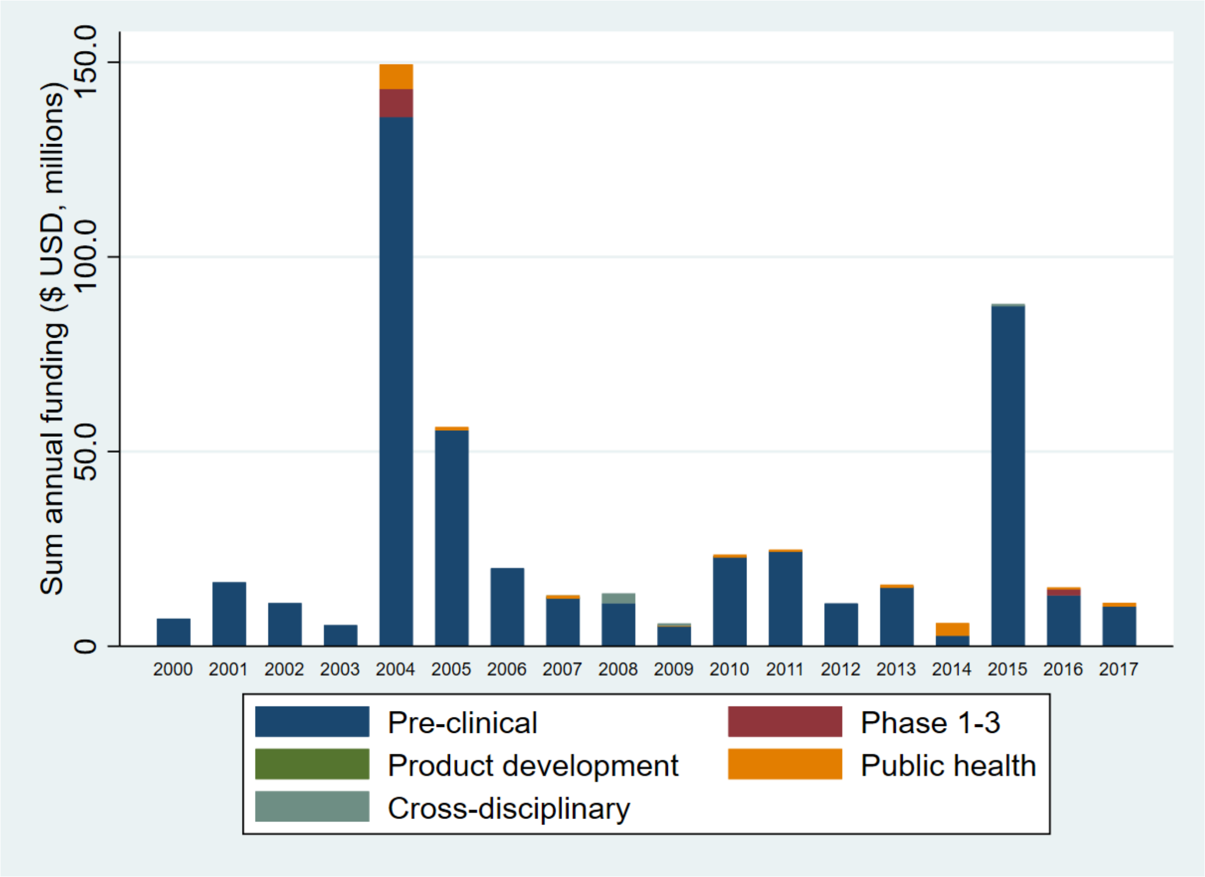

Appendix III – Research investments related to Coronavirus 2000-2017, by year and type of science

Appendix IV – R&D investment related to Ebola virus 2000-2017

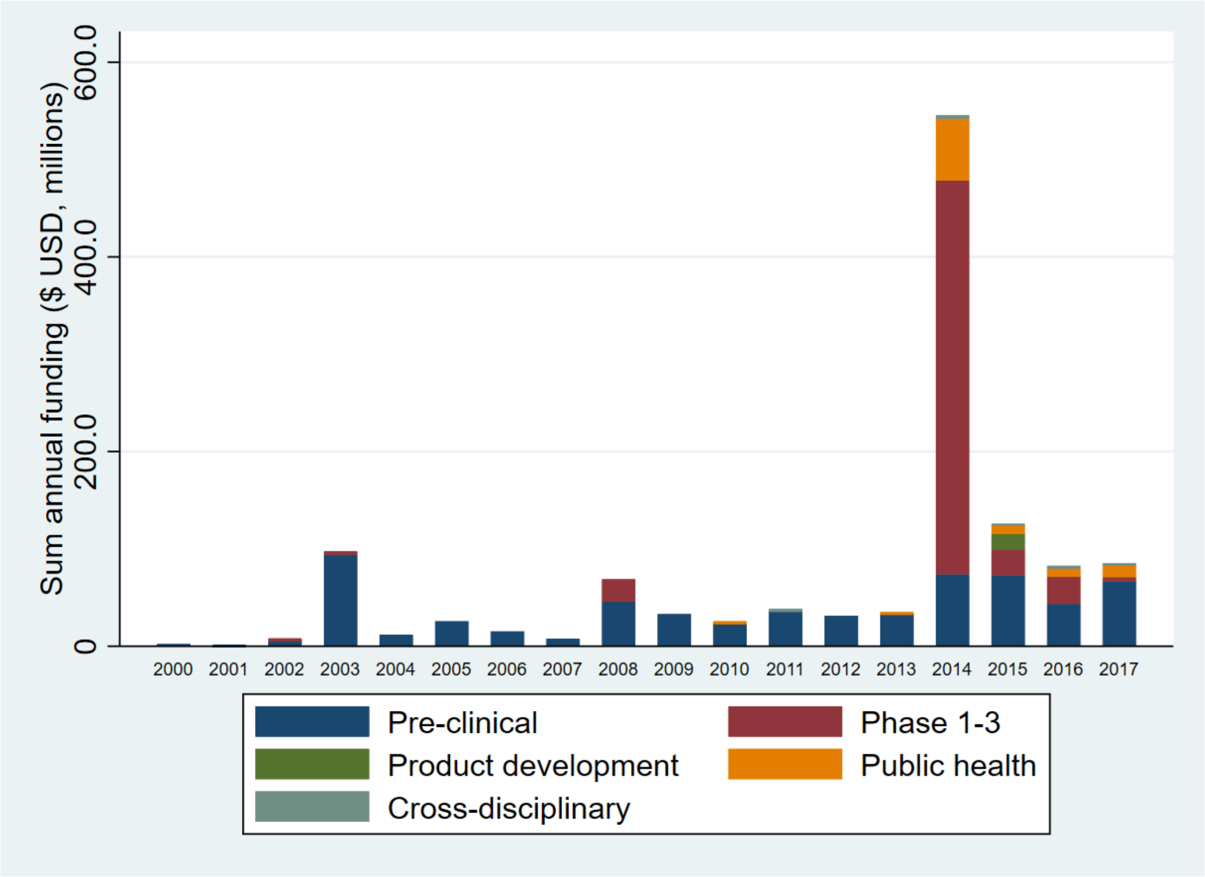

Appendix IV – Research investments related to Ebola virus 2000-2017, by year and type of science

Appendix V – R&D investment related to Zika virus 2000-2017

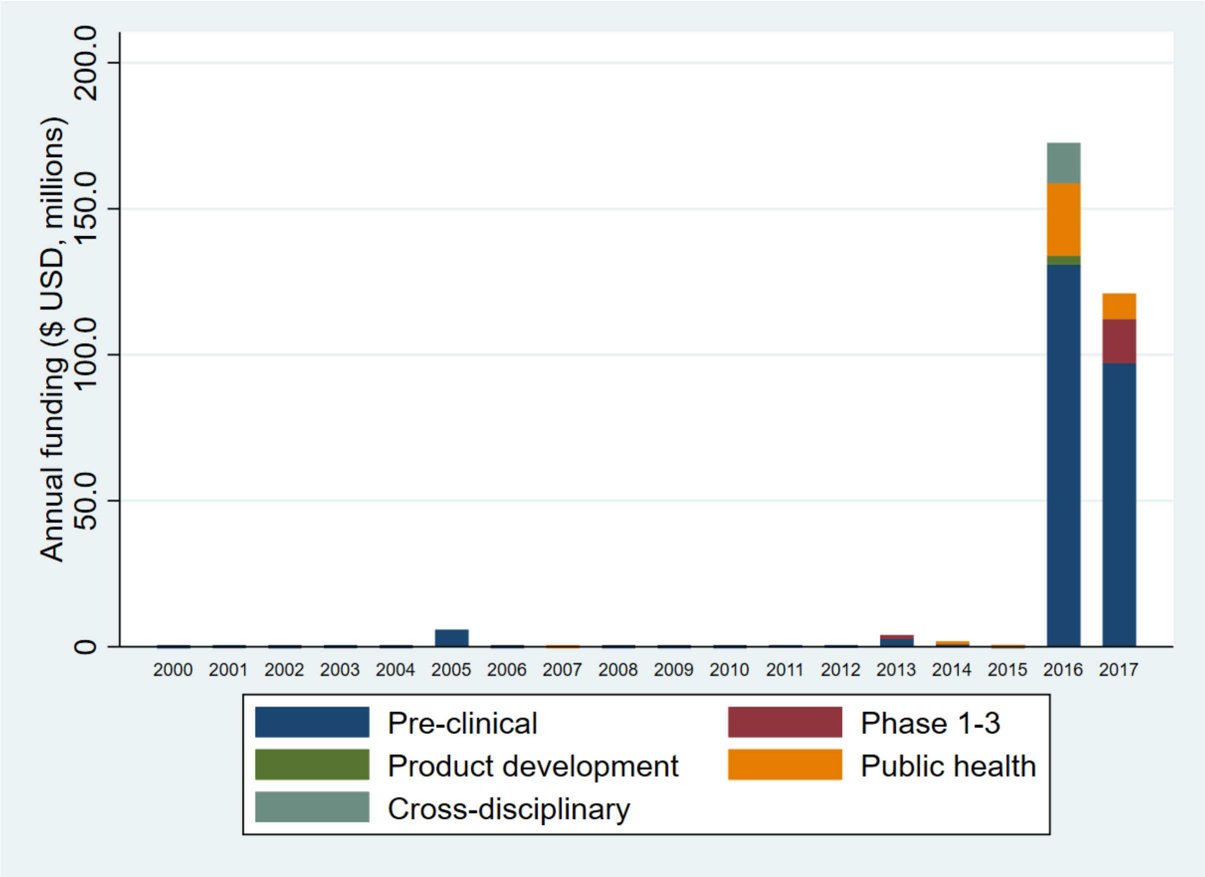

Appendix V – Research investments related to Zika virus 2000-2017, by year and type of science

Appendix VI – Donor investments by year

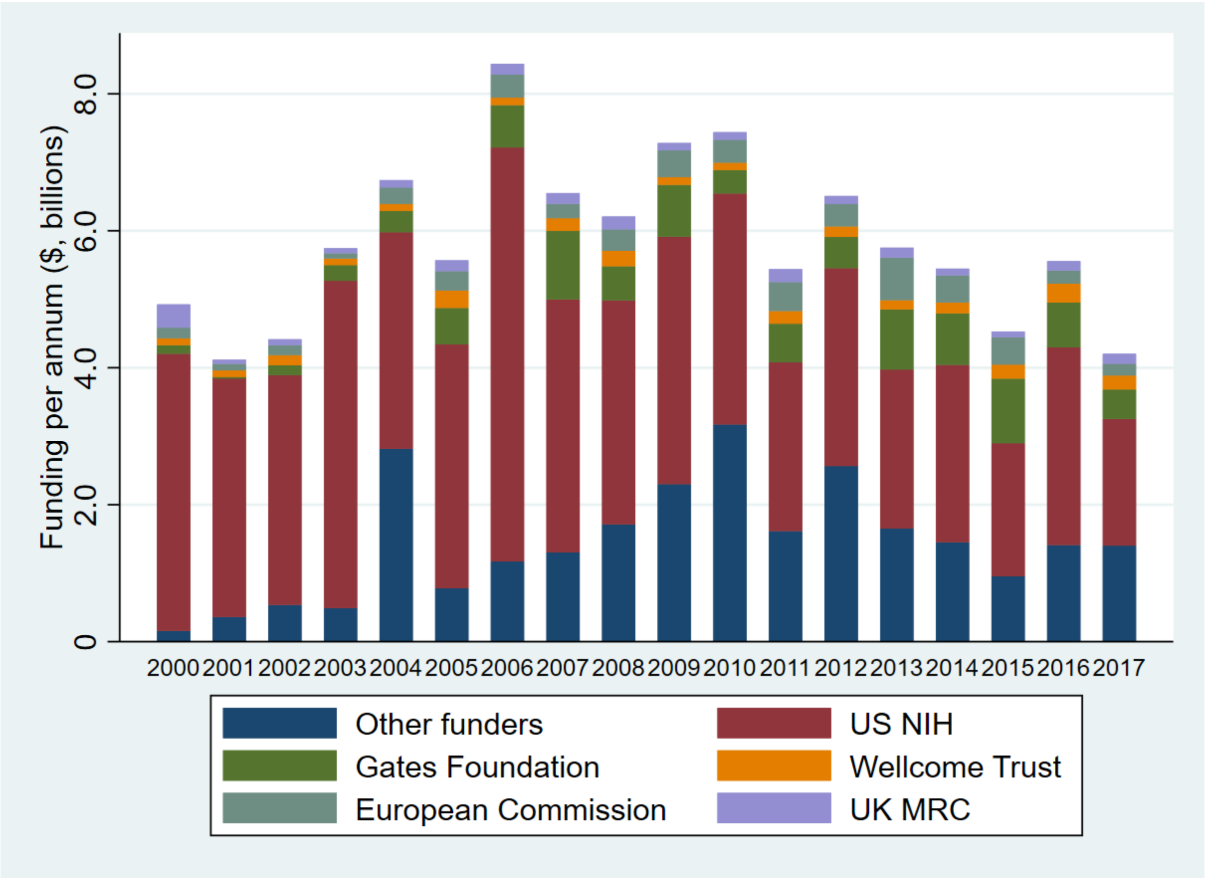

Appendix VII - Donor investments, proportion of funding by year

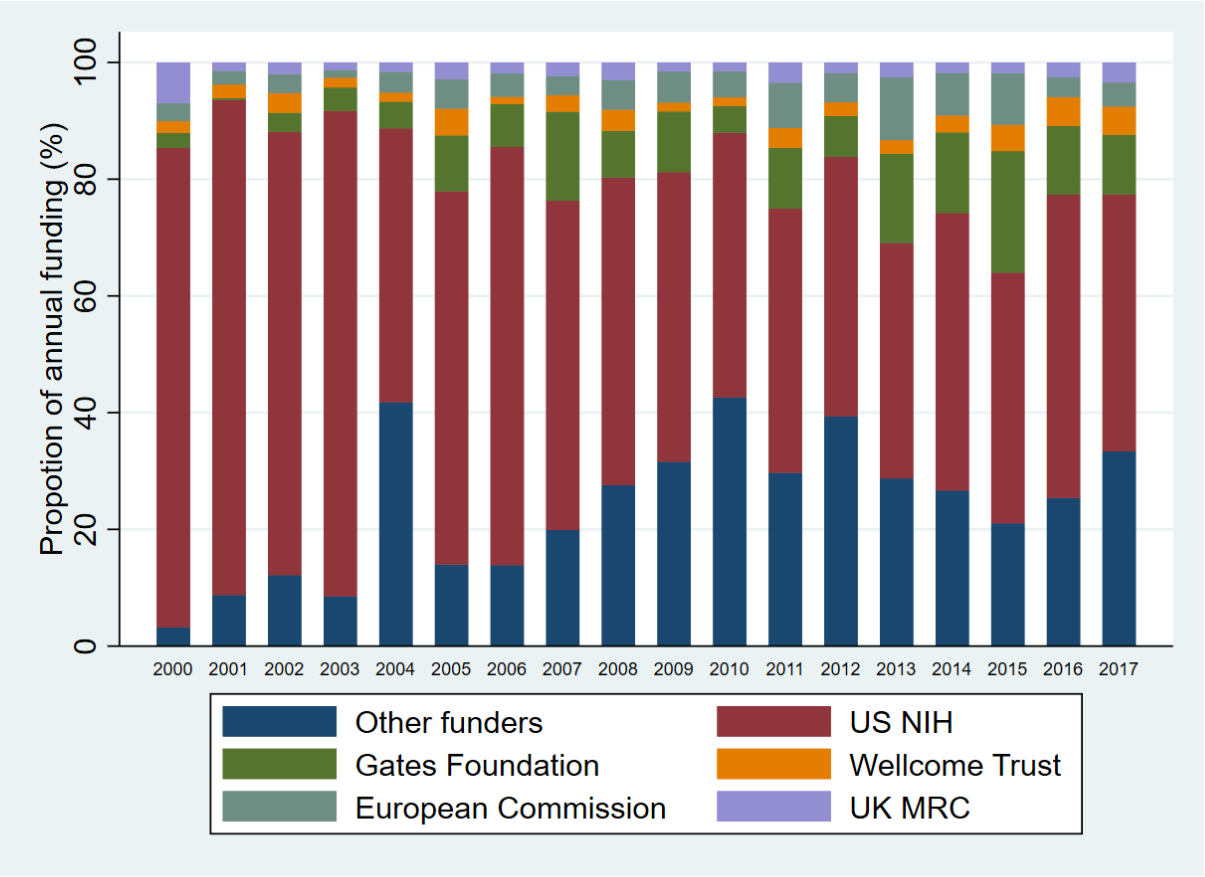

Appendix VIII - Research pipeline for HIV/AIDS, malaria, tuberculosis and pneumonia 2000-2017

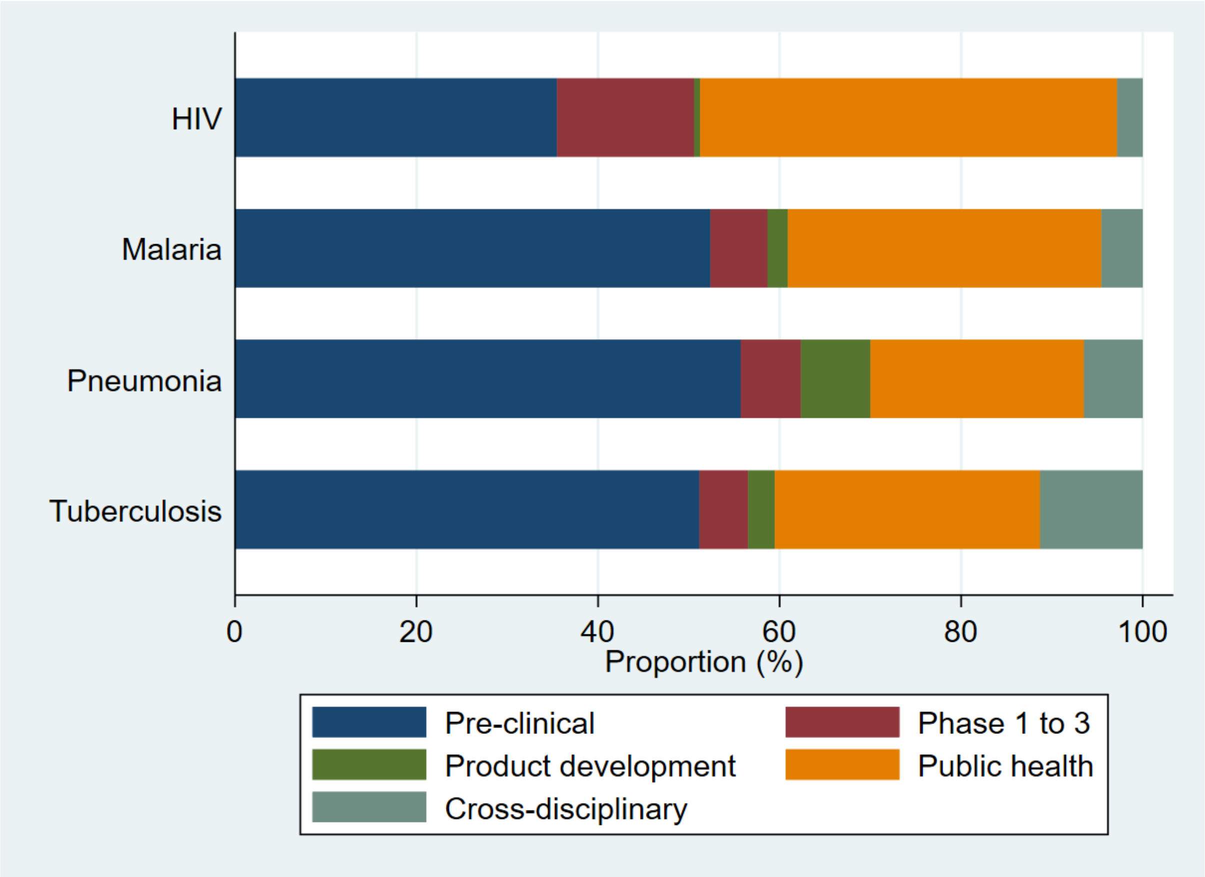

Appendix IX. Investment for selected infectious diseases compared with burden of disease

Disease	Funding	DALYs in 2017	Funding per 2017 DALY	Ranking of Funding per 2017 DALY
African trypanosomiasis	$769 373 088	78 990	$9 740	1
Genital herpes	$767 350 984	247 449	$3 101	2
Leprosy	$72 846 615	31 513	$2 312	3
Chlamydial infection	$748 071 028	355 096	$2 107	4
Chagas disease	$359 090 978	232 143	$1 547	5
Gonococcal infection	$312 583361	303 103	$1 031	6
Leishmaniasis	$754 241 255	774 211	$974	7
HIV/AIDS	$42 024 500 000	54 446 184	$772	8
Trichomoniasis	$139 661 501	242 814	$575	9
Dengue	$1 156 591 953	2 922 630	$396	10
Sexually transmitted infections excluding HIV	$3 736 444 164	11 473 757	$326	11
Lymphatic filariasis	$402 854 250	1 363 953	$295	12
Yellow fever	$89 354 426	314 002	$285	13
Schistosomiasis	$393 057 872	1 431 447	$275	14
Trachoma	$79 259 634	302 919	$262	15
Rabies	$106 234 137	633 806	$168	16

Disease	Funding	DALYs in 2017	Funding per 2017 DALY	Ranking of Funding per 2017 DALY
Tuberculosis	$7,026,261,356	44 997 359	$156	17
Hepatitis C	$2 414 606 412	15 598 250	$155	18
Varicella and herpes zoster	$153 917 628	1 144 435	$134	19
Onchocerciasis	$171 685 350	1 342 937	$128	20
Malaria	$5 645 007 933	45 014 578	$125	21
Typhoid and paratyphoid	$967 958 038	9 800 988	$99	22
Urinary tract infections	$363 784 033	4 695 291	$77	23
Enteric infections	$6 498 954 989	95 209 183	$68	24
Hepatitis E	$35 854 299	738 508	$49	25
Measles	$308 012 254	8 156 526	$38	26
Hepatitis B	$917 469 643	25 282 942	$36	27
Pneumonia	$3,560 915 407	106 483 431	$33	28
Meningitis	$575 154 455	20 370 870	$28	29
Pertussis	$205 556 273	7 977 284	$26	30
Hepatitis A	$37 511 691	1 497 892	$25	31
Tetanus	$24 110 176	2 449 433	$10	32
Scabies	$42 279961	4 528 672	$9	33
Syphilis	$84 965 600	9 909 025	$9	34
